# Anatomic risk factor for meniscal lesion in association with ACL rupture

**DOI:** 10.1186/s13018-019-1281-z

**Published:** 2019-07-30

**Authors:** Romain Gaillard, Robert Magnussen, Cecile Batailler, Philippe Neyret, Sebastien Lustig, Elvire Servien

**Affiliations:** 1 Department of Orthopaedics, Groupement Hospitalier Nord, Université Lyon 1, 103 Grande rue de la Croix Rousse, 69004 Lyon, France; 2 Department of Orthopaedics, The Ohio State University, 2050 Kenny Rd #3100, Columbus, OH 43210 USA; 3 Univ Lyon, Université Claude Bernard Lyon 1, IFSTTAR, LBMC UMR_T9406, F69622 Lyon, France; 4 Univ Lyon, Université Claude Bernard Lyon 1, LIBM, Villeurbanne, 69100 France; 5 OSU Sports Medicine Research Institute, The Ohio State University, 2050 Kenny Rd #3100, Columbus, OH 43210 USA

**Keywords:** ACL, Meniscal tear, CT scan, Anatomy

## Abstract

**Background:**

To assess anatomic risk factors for meniscal lesions in association with acute ACL rupture. The primary hypothesis was that tibiofemoral anatomic measures will be different in those with and without concomitant meniscus tears.

**Methods:**

A retrospective review of patients who underwent acute ACL reconstruction in the department was performed. All patients underwent a postoperative CT scan. The concavity and/or convexity on the femur and the tibia were measured by two blinded observers on the sagittal plane with different ratios, and these measures were compared in patients with and without meniscus tears in each compartment. Intra- and inter-rater reliabilities were assessed.

**Results:**

Four hundred twelve patients (268 males and 144 females) were included from October 2012 to February 2015. One hundred sixty-seven patients had a medial meniscal tear (119 males/48 females), and 100 had a lateral meniscal tear (80 males/20 females). The mean time from injury to surgery was 3 months. The average ICC for all measurements was 0.87 (range 0.82–0.98) indicating good reliability. The medial femoral condyle was noted to be significantly longer than the medial tibial plateau in the sagittal plane in patients with a medial meniscal tear (*p* = 0.04), and the lateral femoral condyle was noted to be significantly longer than the lateral tibial plateau in the sagittal plane in patients with a lateral meniscal tear (*p* <  0.001). In addition, a less convex lateral tibial plateau was statistically correlated with a higher risk of lateral meniscal tear (*p* = 0.001).

**Conclusions:**

A greater anteroposterior length of the medial/lateral femoral condyle relative to the medial/lateral tibial plateau is associated with an increased risk of meniscal lesions in association with acute ACL rupture. The lateral compartment in the male population appears to be the most at risk.

**Trial registration:**

Retrospectively registered on May 12, 2016 (CPP sud-est II CAL n°2016-037)

## Background

There is a relatively high incidence of concurrent meniscus tears (40% to 68%) in association with anterior cruciate ligament (ACL) rupture [1–4], with the lateral meniscus most frequently involved in acute ACL injuries [5, 6]. The presence of these associated meniscus tears increases the risk of subsequent degenerative change, and it is desirable to repair and preserve the meniscus whenever possible [7].

Previous work has evaluated patient and injury factors associated with meniscus injury in the setting of ACL tears. Male sex, age less than 30 years, and injury during contact sports is associated with medial meniscus injury [8], while increased age has been associated with an increased risk of lateral meniscus injury [9]. There has been relatively little work evaluating the association between anatomical factors and the risk of concomitant meniscus injury.

There is, however, an abundance of research investigating anatomic risk factors for isolated ACL rupture (excessive tibial slope [10–12], intercondylar notch width [13, 14], lateral morphology of the knee [15–17]). Limited prior work evaluating risk factors associated with meniscus pathology has suggests decreased femorotibial congruency, excessive tibial rotation, and a discrepancy between femoral condyle and the tibial plateau lengths may play a role [18–20].

The aim of this study is to identify correlations between the bony morphology analyzed on CT scan and the presence of meniscal lesions in association with acute ACL rupture.

It is hypothesized that sagittal plane differences in condylar and plateau length as well as concavity are associated with meniscus status at the time of ACL reconstruction.

## Methods

### Subjects

Subjects were identified retrospectively from a prospective database of 958 patients who underwent ACL reconstruction (ACL-R) between October 2012 and February 2015 at the same institution. CT scans were performed postoperatively, as a routine examination when possible (initially for assessment of the positioning of femoral and tibial tunnels after ACL-R). The study protocol was reviewed and approved by an institutional review board.

Inclusion criteria were:

- Patients who underwent acute ACL-R (within 6 months of injury) without concomitant procedures other than meniscal procedures
- Age 18 to 60 years
- No previous surgery
- Documented date and mechanism of injury
- Post-operative CT scan completed

Exclusion criteria were:

- Revision ACL-R surgery
- Previous meniscal surgery
- Associated fractures other than Segond fractures
- Post-traumatic deformities
- Associated surgery (osteotomies, other ligament repairs or reconstructions)
- ACL-R performed more than 6 months after the initial injury

After applying the above criteria, 412 patients who had an acute ACL-R with or without associated medial or lateral meniscus lesion were identified (Fig. 3).

Demographic and injury data were recorded. All patients underwent a pre-operative clinical exam with the IKDC scoring system [21, 22]. Athletic activities were graded by the Tegner activity scale [23]. During ACL-R surgery under arthroscopy, data were collected regarding the presence of medial and/or lateral meniscus lesions according to the ISAKOS classification system [24].

### CT scan measurements protocol

All CT scans were standardized by to ensure that the axial, coronal, and sagittal reconstructed images were orthogonal to the posterior femoral condyles and that the articular surfaces could be accurately and precisely visualized. Protocol from a previous study from the same institution published by Schneider et al. [25] was used. All examinations were carried out in the same institutional radiology department.

Images were transferred in DICOM (Digital Imaging and Communications in Medicine) format from the institution’s electronic PACS (picture archiving and communication system) software (Centricity; GE Healthcare, Waukesha, Wisconsin) to OrisiX. The measuring instrument in the software was used to define angles to within 0.1° and lengths to within 0.1 mm for measured distances.

Two independent observers performed all measurements.

### Sagittal geometry evaluation

**Fig. 1:**
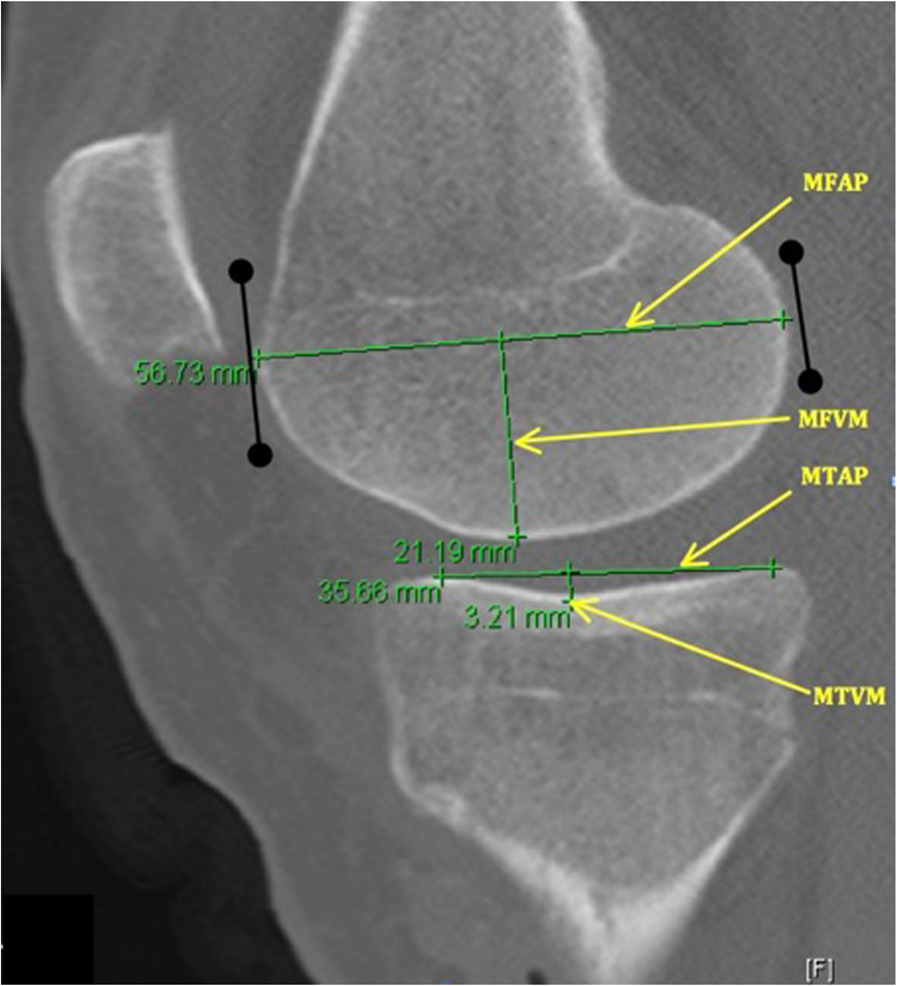
Measurements on a sagittal CT reconstruction of the medial compartment. MFAP: medial femoral condyle antero-posterior length. MFVM: medial femoral vertical maximum of the curvature. MTAP: medial tibial condyle antero-posterior length. MTVM: medial tibial plateau vertical maximum of the curvature

**Fig. 2:**
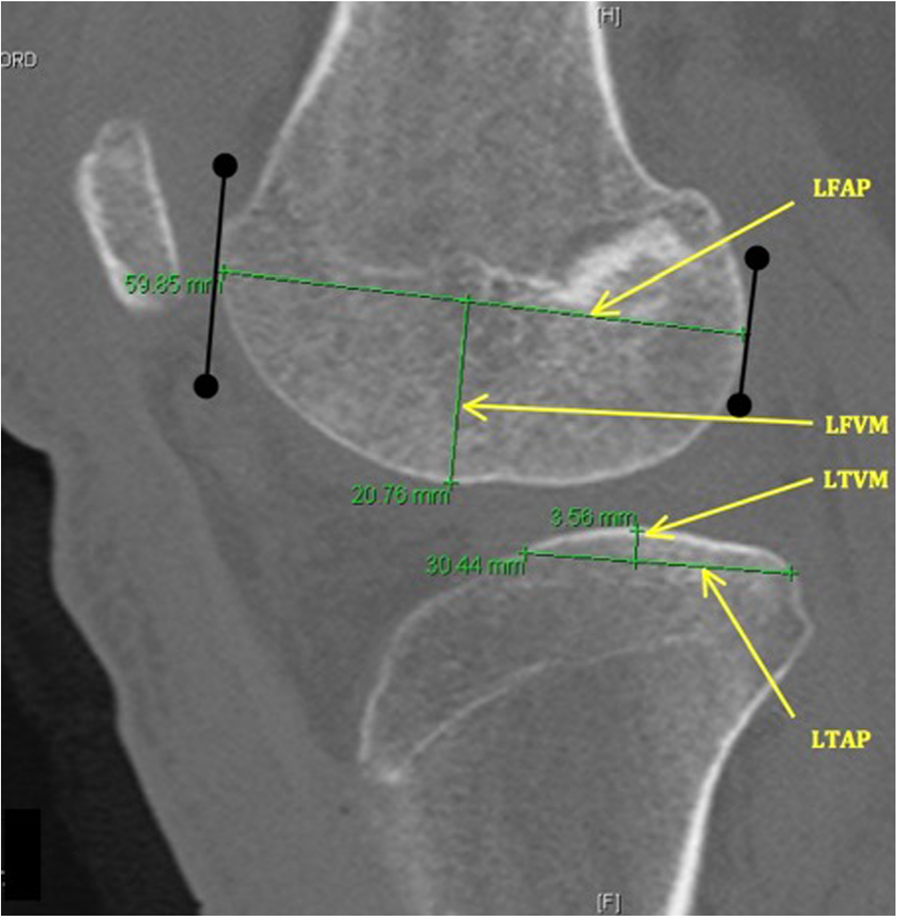
Measurements on a sagittal CT reconstruction of the lateral compartment. LFAP: lateral femoral condyle antero-posterior length. LFVM: lateral femoral vertical maximum of the curvature. LTAP: lateral tibial condyle antero-posterior length. LTVM: lateral tibial plateau vertical maximum of the curvature

All measurements used the method of Schneider et al. [25], inspired by the work of Wahl et al. [17] with simplification of the technique in order to describe the convexities or concavities of the articular surfaces (Figs. 1 and 2).

The maximum femoral condyle antero-posterior length was measured for each lateral (LFAP) or medial (MFAP) compartment on a sagittal reconstruction. It was defined as the greatest distance between the anterior and posterior articular surfaces of each condyle.

The maximum tibial plateau antero-posterior length of the lateral (LTAP) and medial (MTAP) tibial plateaus was defined by the anatomy of each plateau (top of the concavity for the medial tibial plateau, base of the convexity for the lateral one) and was measured as the distance between the most anterior and posterior margins of the tibial plateau subchondral bone.

Due to differences in patient size, the ratios of medial and lateral anteroposterior femoral length to tibial length (MFAP to MTAP and LFAP to LTAP) were calculated to assess whether the size of the tibia relative to femur was associated with meniscus injury regardless of patient size. It defined lateral sagittal femoro-tibial ratio (LSR) and medial sagittal femoro-tibial ratio (MSR) as a description of the discrepancy sagittal length of the femur on the tibia.

For each of the maximal anterior–posterior lengths previously described, a perpendicular line was created between the line used to measure the length of the surface and the most distant subchondral bone. The length of this line was defined at the height of each osseous element: lateral tibial plateau vertical maximum of the curvature (LTVM), medial tibial plateau vertical maximum of the curvature (MTVM), lateral femoral vertical maximum of the curvature (LFVM), and medial femoral vertical maximum of the curvature (MFVM).

The concavity or convexity of each element was described by the ratio of this vertical line to its anterior–posterior length, with low values signifying increased convexity or concavity:

- MTAP to MTVM for assessment of the medial tibial plateau concavity (MTC)
- LTAP to LTVM for assessment of the lateral tibial plateau convexity (LTC)
- MFAP to MFVM and LFAP to LFVM for assessment of the lateral and medial femoral condyles convexity (LFC and MFC)

### Statistical analysis

The two-sample Student *t* test was used to determine whether measurements differed significantly based on meniscus status at ACL reconstruction. Because lateral meniscus tears were more frequent in males compared to females, the analysis was repeated while stratifying based on sex. Categorical data were compared using the chi-squared test. All continuous data were found to follow a normal distribution. To assess the reproducibility of the different measurements, intra-class correlations (ICC) were calculated. An ICC value greater than 0.9 was considered excellent, and a value between 0.9 and 0.8 was considered good [26]. Intra-observative variability was assessed by the same surgeon re-measuring all CT scans. Another independent surgeon measured CT scans too, to determine inter-observer variability.

Statistical analysis was performed with the use of SAS software (version 9.2; SAS Institute, Cary, NC). A *p* value of less than 0.05 was considered to be significant.

## Results

**Fig. 3:**
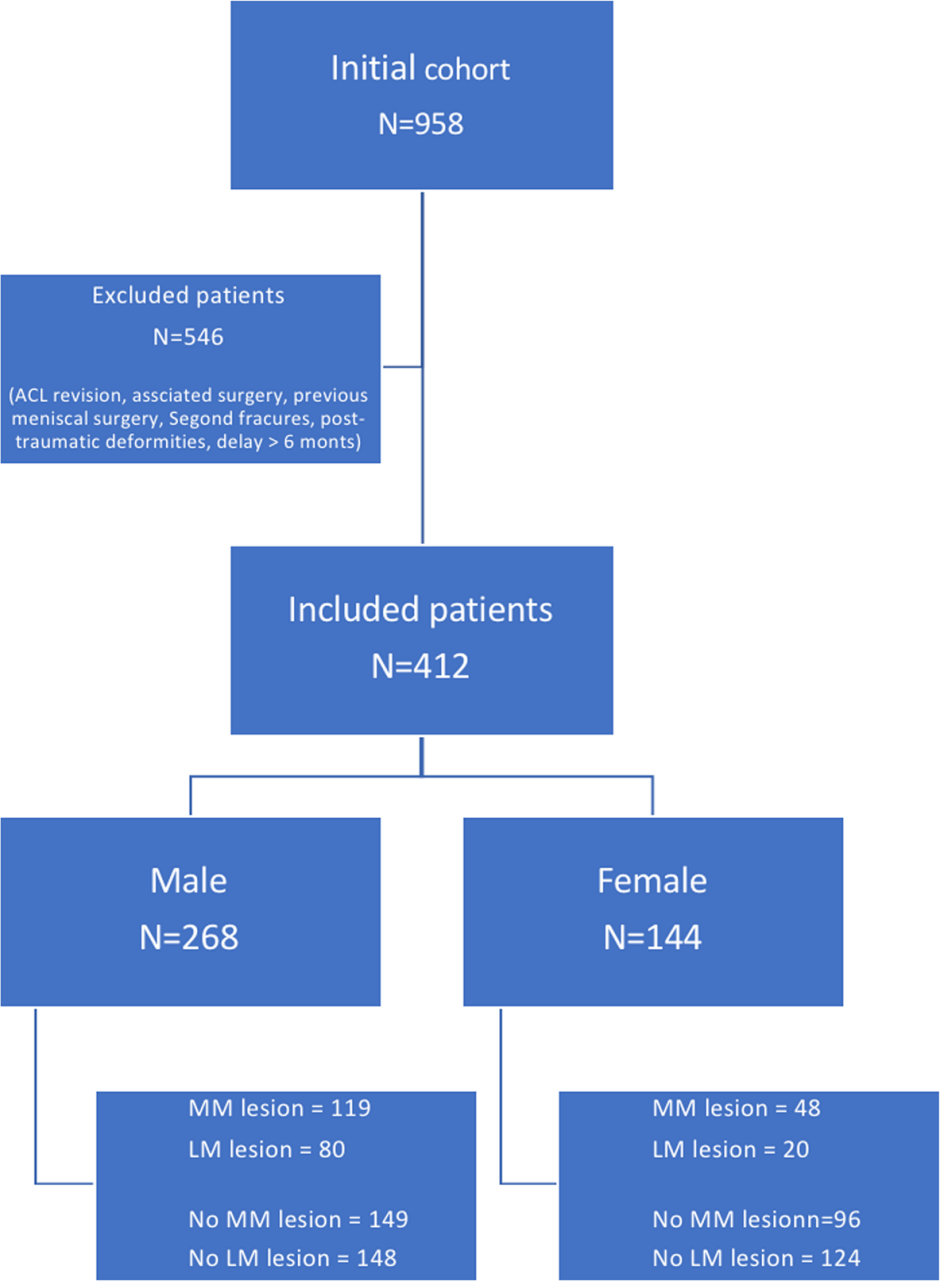
Flowchart

There were 412 patients meeting the inclusion criteria: 268 (65%) men and 144 (35%) women. One hundred nineteen men (44%) and 48 females (33%) had a medial meniscal (MM) lesion, while 80 men (30%) and 20 females (14%) had a lateral meniscal (LM) lesion (Fig. 3). The injury mechanism was related to sports in 93% of cases (soccer and skiing represented the majority of injuries). Other causes were motor vehicle accidents (1%), domestic accidents (4%), and work accidents (2%).

Characteristics of the populations with and without meniscal lesions are described in Table 1. Patients with MM lesions were older (34 vs 30; *p* <  0.001) and had a higher body mass index (BMI) (24.5 vs 23.7; *p* = 0.02).

**Table 1:** Demographics data of patients with and without medial and lateral meniscal lesions

	Mean ± SD	Minimum	Maximum	*p*
Age at surgery (years)				
With MM lesion (*n* = 167)	34.4 ± 1.8	15.4	62	< 0.001
No MM lesion (*n* = 245)	30.1 ± 1.3	15.7	58.1	
With LM lesion (*n* = 100)	31.3 ± 2.2	16.3	51.1	0.6
No LM lesion (*n* = 312)	32 ± 1.3	15.4	62	
BMI (kg/m^2^)				
With MM lesion (*n* = 167)	24.5 ± 0.5	17.5	37.3	0.02
No MM lesion (*n* = 245)	23.7 ± 0.4	17.7	37.6	
With LM lesion (*n* = 100)	24.2 ± 0.6	17.6	32.4	0.6
No LM (*n* = 312)	24 ± 0.4	17.5	37.6	
Tegner activity score				
With MM lesion (*n* = 167)	6.8 ± 0.2	2	9	0.4
No MM lesion (*n* = 245)	6.9 ± 0.1	2	10	
With LM lesion (*n* = 100)	6.9 ± 0.2	2	10	0.6
No LM lesion (*n* = 312)	6.8 ± 0.1	2	9	
Time from injury to surgery (weeks)				
With MM lesion (*n* = 167)	15.1 ± 2.7	0.5	123.9	0.08
No MM lesion (*n* = 245)	9.3 ± 2.2	0.4	155.8	
With LM lesion (*n* = 100)	9.1 ± 3.3	0.4	154	0.3
No LM lesion (*n* = 312)	11.8 ± 2.6	0.6	155.8	
IKDC score	B	C	D	
With MM lesion (*n* = 167)	12 (7.2%)	132 (79%)	23 (13.8%)	0.2
No MM lesion (*n* = 245)	22 (9%)	203 (82.9%)	20 (8.1%)	
With LM lesion (*n* = 100)	4 (4%)	81 (81%)	15 (15%)	0.02
No LM lesion (*n* = 312)	30 (9.6%)	254 (81.4%)	28 (9%)	

The reproducibility of the measurements after reconstruction of the tibial and femoral bone segments in dedicated image processing software was good to excellent, with ICCs between 0.82 and 0.98 (Table 2). The average ICC for all measurements was 0.87.

**Table 2:** Intra- and inter-observer reliability of articular geometry

	Mean ± SD	ICC (95% CI)
Medial sagittal femoro-tibial ratio (MSR)		
Reviewer 1		
Analysis 1	1.59 ± 0.02	0.94 (0.91–0.98)
Analysis 2	1.61 ± 0.05	
Reviewer 2	1.62 ± 0.03	0.92 (0.87–0.94)
Lateral sagittal femoro-tibial ratio (LSR)		
Reviewer 1		
Analysis 1	2.11 ± 0.04	0.98 (0.95–0.99)
Analysis 2	2.11 ± 0.03	
Reviewer 2	2.14 ± 0.04	0.88 (0.82–0.93)
Medial tibial plateau concavity (MTC)		
Reviewer 1		
Analysis 1	13.6 ± 0.91	0.89 (0.84–0.92)
Analysis 2	13.2 ± 0.73	
Reviewer 2	13.4 ± 0.65	0.92 (0.87–0.95)
Lateral tibial plateau convexity (LTC)		
Reviewer 1		
Analysis 1	16.07 ± 1.38	0.82 (0.77–0.85)
Analysis 2	15.35 ± 0.99	
Reviewer 2	18.64 ± 1.83	0.85 (0.81–0.92)
Medial femoral condyle convexity (MFC)		
Reviewer 1		
Analysis 1	2.89 ± 0.02	0.98 (0.95–0.99)
Analysis 2	2.9 ± 0.05	
Reviewer 2	2.89 ± 0.02	0.95 (0.9–0.98)
Lateral femoral condyle convexity (LFC)		
Reviewer 1		
Analysis 1	2.94 ± 0.02	0.97 (0.93–0.99)
Analysis 2	2.95 ± 0.04	
Reviewer 2	2.97 ± 0.03	0.88 (0.83–0.92)

In the global population (Table 3), patients with MM lesions were noted to have a higher mean MSR (1.58 ± 0.02) than those without (1.55 ± 0.02) (*p* = 0.04). Patients with LM lesions were noted to have a higher mean LSR (2.16 ± 0.04) than those without (2.06 ± 0.03) (*p* <  0.001). The mean LTC was higher in patients with LM lesions (15.37 ± 0.99) than those without (19.08 ± 1.99) (*p* = 0.001).

**Table 3:** Anatomical characteristics in relation on medial and lateral meniscal tears

	Mean ± SD	Minimum	Maximum	*p*
Medial sagittal femoro-tibial ratio (MSR)				
With MM lesion (*n* = 167)	1.58 ± 0.02	0.61	2.05	0.04
No MM lesion (*n* = 245)	1.55 ± 0.02	1.06	2	
With LM lesion (*n* = 100)	1.61 ± 0.05	0.67	2.42	0.7
No LM lesion (*n* = 312)	1.6 ± 0.03	0.61	2.43	
Lateral sagittal femoro-tibial ratio (LSR)				
With MM lesion (*n* = 167)	2.09 ± 0.04	0.51	2.86	0.8
No MM lesion (*n* = 245)	2.09 ± 0.03	1	2.62	
With LM lesion (*n* = 100)	2.16 ± 0.04	1.59	2.52	< 0.001
No LM lesion (*n* = 312)	2.06 ± 0.03	0.51	2.86	
Medial tibial plateau concavity (MTC)				
With MM lesion (*n* = 167)	13.72 ± 0.93	2.46	46.16	0.4
No MM lesion (*n* = 245)	13.2 ± 0.71	2.54	40.21	
With LM lesion (*n* = 100)	13.22 ± 1	2.58	26.92	0.7
No LM lesion (*n* = 312)	13.47 ± 0.68	2.46	46.16	
Lateral tibial plateau convexity (LTC)				
With MM lesion (*n* = 167)	16.04 ± 1.38	3.01	63.02	0.5
No MM lesion (*n* = 245)	16.66 ± 1.27	3.33	72.87	
With LM lesion (*n* = 100)	15.37 ± 0.99	3.01	72.87	0.001
No LM lesion (*n* = 312)	19.08 ± 1.99	6.32	63.02	
Medial femoral condyle convexity (MFC)				
With MM lesion (*n* = 167)	2.89 ± 0.03	2.34	3.46	0.6
No MM lesion (*n* = 245)	2.9 ± 0.02	2.38	3.66	
With LM lesion (*n* = 100)	2.9 ± 0.04	2.47	11.19	0.8
No LM lesion (*n* = 312)	2.89 ± 0.02	1.86	48.46	
Lateral femoral condyle convexity (LFC)				
With MM lesion (*n* = 167)	2.94 ± 0.03	1.45	3.44	0.2
No MM lesion (*n* = 245)	2.97 ± 0.02	2.54	13.35	
With LM lesion (*n* = 100)	2.97 ± 0.04	2.5	3.66	0.3
No LM lesion (*n* = 312)	2.95 ± 0.02	1.58	13.35	

In females (Table 4), there were no statistically significant differences in anatomic characteristics based on whether medial or lateral meniscus tears are present.

**Table 4:** Anatomical characteristics in relation to medial and lateral meniscal tears, in females

	Mean ± SD	Minimum	Maximum	*p*
Medial sagittal femoro-tibial ratio (MSR)				
With MM lesion (*n* = 48)	1.59 ± 0.03	1.3	1.97	0.2
No MM lesion (*n* = 96)	1.54 ± 0.05	0.67	1.81	
With LM lesion (*n* = 20)	1.58 ± 0.15	0.67	2.4	0.5
No LM lesion (*n* = 124)	1.63 ± 0.04	1.3	2.33	
Lateral sagittal femoro-tibial ratio (LSR)				
With MM lesion (*n* = 48)	2.09 ± 0.09	0.51	2.52	0.7
No MM lesion (*n* = 96)	2.11 ± 0.04	1.64	2.62	
With LM lesion (*n* = 20)	2.16 ± 0.1	1.83	2.52	0.09
No LM lesion (*n* = 124)	2.09 ± 0.04	0.51	2.62	
Medial tibial plateau concavity (MTC)				
With MM lesion	13.72 ± 1.8	2.55	35.37	0.6
No MM lesion	13.04 ± 1.12	2.62	40.17	
With LM lesion	13.29 ± 2.2	2.83	20.9	0.9
No LM lesion	13.25 ± 1.06	2.55	40.17	
Lateral tibial plateau convexity (LTC)				
With MM lesion	14.18 ± 2.46	3.01	54.5	0.6
No MM lesion	13.48 ± 1.24	3.33	42.74	
With LM lesion	13.19 ± 2.79	7.92	30.91	0.7
No LM lesion	13.8 ± 1.27	3.01	54.5	
Medial femoral condyle convexity (MFC)				
With MM lesion	2.9 ± 0.06	2.34	3.38	0.6
No MM lesion	2.93 ± 0.04	2.52	3.66	
With LM lesion	3.33 ± 0.82	2.52	11.19	0.3
No LM lesion	2.92 ± 0.04	1.86	3.66	
Lateral femoral condyle convexity (LFC)				
With MM lesion	2.97 ± 0.06	1.45	3.34	0.4
No MM lesion	3 ± 0.04	2.6	3.48	
With LM lesion	3.03 ± 0.09	2.69	3.48	0.7
No LM lesion	3.06 ± 0.13	1.58	11.01	

In males (Table 5), patients with LM lesions were noted to have a higher mean LSR (*p* <  0.001) and higher mean LTC (*p* = 0.004) than those.

**Table 5:** Anatomical characteristics in relation to medial and lateral meniscal tears, in males

	Mean ± SD	Minimum	Maximum	*p*
Medial sagittal femoro-tibial ratio (MSR)				
With MM lesion (*n* = 119)	1.54 ± 0.02	1.06	2	0.5
No MM lesion (*n* = 114)	1.53 ± 0.03	0.61	2.05	
With LM lesion (*n* = 80)	1.62 ± 0.06	1.28	2.42	0.2
No LM lesion (*n* = 183)	1.58 ± 0.03	0.61	2.43	
Lateral sagittal femoro-tibial ratio (LSR)				
With MM lesion (*n* = 119)	2.08 ± 0.04	1.67	2.86	0.9
No MM lesion (*n* = 114)	2.08 ± 0.04	1	2.62	
With LM lesion (*n* = 80)	2.16 ± 0.04	1.59	2.5	< 0.001
No LM lesion (*n* = 183)	2.05 ± 0.03	1	2.86	
Medial tibial plateau concavity (MTC)				
With MM lesion (*n* = 119)	13.71 ± 1.09	2.46	46.16	0.6
No MM lesion (*n* = 114)	13.31 ± 0.92	2.54	40.21	
With LM lesion (*n* = 80)	13.2 ± 1.13	2.58	26.92	0.6
No LM lesion (*n* = 183)	13.61 ± 0.88	2.46	46.16	
Lateral tibial plateau convexity (LTC)				
With MM lesion (*n* = 119)	16.76 ± 1.65	6.32	63.02	0.2
No MM lesion (*n* = 114)	18.35 ± 1.73	7.02	72.87	
With LM lesion (*n* = 80)	16.4 ± 1.4	7.02	72.87	0.004
No LM lesion (*n* = 183)	20.34 ± 2.3	6.32	63.02	
Medial femoral condyle convexity (MFC)				
With MM lesion (*n* = 119)	3.36 ± 0.77	2.43	3.46	0.2
No MM lesion (*n* = 114)	2.88 ± 0.03	2.38	3.34	
With LM lesion (*n* = 80)	2.89 ± 0.04	2.47	3.34	0.3
No LM lesion (*n* = 183)	3.18 ± 0.49	2.38	48.46	
Lateral femoral condyle convexity (LFC)				
With MM lesion (*n* = 119)	2.94 ± 0.03	2.5	3.44	0.3
No MM lesion (*n* = 114)	3.02 ± 0.14	2.54	13.35	
With LM lesion (*n* = 80)	2.96 ± 0.04	2.5	3.66	0.6
No LM lesion (*n* = 183)	3.99 ± 0.11	2.53	13.35	

## Discussion

The most important findings of this study were that increased anteroposterior compartment ratios were associated with increased risk of meniscus tears of the medial and lateral sides of the knee, while lateral meniscus tears are also associated with increased lateral tibial plateau convexity.

Final findings about discrepancy of the femur on the tibia are close but not strictly comparable to the work of Wahl et al. [17] and Bozkurt et al. [18] that suggested an association between meniscus tears and femorotibial compartment congruency in patients with acute ACL ruptures, defined by a less concave medial tibial plateau articulating with a more convex medial femoral condyle.

These conclusions are to be qualified according to the sex of the patient and the lateralization of the meniscal lesion. Indeed, in men, there is a strong association between lateral meniscus injury and anteroposterior discrepancy of the femoral condyle on the tibial plateau, associated with an increased convexity of the lateral tibial plateau. These findings are not found in women, for whom no anatomical risk factor is statistically present.

Associations between medial congruency and the presence of MM tears were generally weaker than on the lateral side and did not vary based on sex. Indeed, the association between medial femorotibial discrepancy and LM tears was found only for the overall cohort, perhaps reflecting a lack of power in the study to detect this medial anatomic risk factor for distinct male and female populations.

Increased mismatch of the femoral condyle and tibia could influence meniscus load and thus injury risk by two potential mechanisms. First, a relatively smaller tibial plateau would be expected to see increased pressure as load transfer is limited to a smaller area—potentially increasing load on the meniscus. In a related study, Suganuma et al. [20] found that increased medial femoral condylar length was a risk factor for non-healing of medial meniscal lesions in cases of isolated medial meniscus repair—possibly due to a similar mechanism of increased compartment motion and increased strain on the repaired meniscus.

The demographic and clinical analysis of the initial cohort showed a significant association between the existence of an MM lesion and a higher age, as well as an increased BMI. Age as a risk factor for meniscal injury is elsewhere often found in large meta-analyses of meniscal lesions [27, 28]. Tearing of MM seems to be significantly more common in sport-related trauma as shown in a recent study of Pezeshki et al. [29].

In regard to the frequency of meniscus tears by sex, it was found an increased incidence of lateral meniscus tears in males (29%) compared to females (14%) (*p* = 0.004). In their study, Feucht et al. [8] also noted that male sex was a risk factor for lateral meniscus tear in association with an ACL rupture. Similarly, Hede et al. [30] showed the incidence of isolated meniscus tears to be two times larger in males relative to females in their epidemiological study.

One reason for this difference in incidence of lateral meniscus tears based on sex may be differences in bony anatomy. In males, we showed a strong associate between lateral compartment incongruence (increase of the lateral plateau convexity and anteroposterior femorotibial discrepancy) and the presence of a LM tear, but such associates were weaker in females.

The clinical application of these results remains to be defined. Nevertheless, it would be interesting to know the existence of anatomical risk factors for meniscal lesions in high-level sports patients with ACL rupture. In fact, the increased risk of associated meniscal lesions would be a sufficient argument to precisely search a meniscal lesion on a MRI or CT scan and to track down it at the time of the reconstruction of the ACL in order to achieve a targeted suture, especially when it existed a longer time from injury than 6 months before surgery as showed by Di Vico et al. [31].

There are several potential weaknesses of this study. First, when measuring the compartment ratios and convexity and concavity, subchondral bone was utilized rather than articular cartilage because CT scans were used in this analysis. Second, the study includes more males than females, reducing available power to detect statistically significant differences in the female group.

Finally, the study’s retrospective nature induces bias concerning the definition of the chronicity of meniscus lesions. It is possible that some meniscus tears pre-existed the ACL and also possible that some meniscus injuries occurred following ACL injury while the patient was ACL-deficient. It is also possible that some meniscus lesions healed prior to treatment of the ACL. Patients with a delay between trauma and surgery of more than 6 months were not included to minimize the existence of later meniscal injury. Some studies have demonstrated the risk of second meniscal tear or degenerative tear due to chronic instability after ACL rupture is quite high 1 year following injury [32–34].

Despite these limitations, the current study shows that a sagittal femoral-tibial discrepancy is a risk factor of meniscal lesions associated with ACL rupture in the corresponding compartment. The lateral compartment in the male population appears to be the most at risk, associated with an increased lateral tibial convexity.

## Conclusions

A greater anteroposterior length of the medial/lateral femoral condyle relative to the medial/lateral tibial plateau is associated with an increased risk of meniscus lesions in association with acute ACL rupture, especially for lateral meniscal injury in male patients.

## Data Availability

The datasets used and/or analyzed during the current study are available from the corresponding author on reasonable request.

## Abbreviations

ACL: Anterior cruciate ligament
ACL-R: Anterior cruciate ligament reconstruction
BMI: Body mass index
DICOM: Digital Imaging and Communications in Medicine
ICC: Intra-class correlations
IKDC: International Knee Documentation Committee
ISAKOS: International Society of Arthroscopy, Knee Surgery and Orthopaedic Sports Medicine
LFAP: Lateral femoral condyle antero-posterior length
LFC: Lateral femoral condyle convexity
LFVM: Lateral femoral vertical maximum of the curvature
LM: Lateral meniscus
LSR: Lateral sagittal femoro-tibial ratio
LTAP: Lateral tibial plateau antero-posterior length
LTC: Lateral tibial plateau convexity
LTVM: Lateral tibial plateau vertical maximum of the curvature
MFAP: Medial femoral condyle antero-posterior length
MFC: Medial femoral condyle convexity
MFVM: Medial femoral vertical maximum of the curvature
MM: Medial meniscus
MSR: Medial sagittal femoro-tibial ratio
MTAP: Medial tibial plateau antero-posterior length
MTC: Medial tibial plateau concavity
MTVM: Medial tibial plateau vertical maximum of the curvature
PACS: Picture archiving and communication system
